# Using leaf spectroscopy and pigment estimation to monitor indoor grown lettuce dynamic response to spectral light intensity

**DOI:** 10.3389/fpls.2022.1044976

**Published:** 2022-11-21

**Authors:** Laura Cammarisano, Jan Graefe, Oliver Körner

**Affiliations:** Next-Generation Horticultural Systems, Leibniz-Institute of Vegetable and Ornamental Crops (IGZ), Großbeeren, Germany

**Keywords:** indoor farming, precision farming, plant performance control, lettuce, leaf optical properties, pigments, blue light

## Abstract

Rising urban food demand is being addressed by plant factories, which aim at producing quality food in closed environment with optimised use of resources. The efficiency of these new plant production systems could be further increased by automated control of plant health and nutritious composition during cultivation, allowing for increased produce value and closer match between plant needs and treatment application with potential energy savings. We hypothesise that certain leaf pigments, including chlorophylls, carotenoids and anthocyanins, which are responsive to light, may be good indicator of plant performance and related healthy compounds composition and, that the combination of leaf spectroscopy and mathematical modelling will allow monitoring of plant cultivation through noninvasive estimation of leaf pigments. Plants of two lettuce cultivars (a green- and a red-leaf) were cultivated in hydroponic conditions for 18 days under white light spectrum in climate controlled growth chamber. After that period, plant responses to white light spectrum (‘W’) with differing blue wavelengths (‘B’, 420 - 450 nm) percentage (15% ‘B15’, and 40% ‘B40’) were investigated for a 14 days period. The two light spectral treatments were applied at photon flux densities (PFDs) of 160 and 240 µmol m^-2^ s^-1^, resulting in a total of four light treatments (160WB15, 160WB40, 240WB15, 240WB40). Chlorophyll *a* fluorescence measurements and assessment of foliar pigments, through destructive (*in vitro*) and non-destructive (*in vivo*) spectrophotometry, were performed at 1, 7 and 14 days after treatment initiation. Increase in measured and estimated pigments in response to WB40 and decrease in chlorophyll:carotenoid ratio in response to higher PFD were found in both cultivars. Cultivar specific behavior in terms of specific pigment content stimulation in response to time was observed. Content ranges of modelled and measured pigments were comparable, though the correlation between both needs to be improved. In conclusion, leaf pigment estimation may represent a potential noninvasive and real-time technique to monitor, and control, plant growth and nutritious quality in controlled environment agriculture.

## 1 Introduction

### 1.1 Semi- and fully- controlled environment agriculture for predictable crop yield and quality

Increased yield and nutritional quality of crop food are currently some of the main targets in horticulture and plant science due to rising world human population. The extreme and unpredictable climate events occurring all around the world threaten field crop production besides reducing crop quality (Maggiore et al., 2020). In greenhouses, the disturbances from the outside climate are minimised compared to open field production, while climate can be controlled to the desired set-point targets only to a certain extend (Gurban and Andreescu, 2018; Choab et al., 2019). The strong impact of solar radiation entering the greenhouse through the transparent cover material is the major driver for short-term greenhouse micro-climate dynamics (Körner and Challa, 2003a), often yielding in growth and quality reduction (Körner and Challa, 2003b). Moreover, the semi-controlled environment of greenhouses is more susceptible to influence from outdoor weather, thus a constant and highly predictable crop yield and quality is difficult to obtain throughout the year (Graamans et al., 2017). A nearly constant and controlled climate can be obtained when totally refraining from energy input by solar radiation in indoor farms. In these closed and fully controlled environments, artificial light substitutes solar radiation and climate can be controlled close to the desired set-points (Kozai, 2013; van Delden et al., 2021). Fluctuation in climate variables as temperature and humidity is avoided, which reduces the risks for several diseases and physiological disorders (Körner and Challa, 2003b). Actively controlling the cultivation light by only applying artificial light, in addition to air conditions, is therefore the key for predictable yield and quality. Since their first applications in indoor farming in the 2000s (Goto, 2012), light emitting diodes (LEDs) allowed their dominance in horticulture over conventional light sources, e.g. high-pressure sodium lamps or fluorescent lamps (Paucek et al., 2020). The fast evolving LED technology enables precision light control (Neo et al., 2022) with highly targeted narrow bandwidths and tuneable intensity (Van Iersel, 2017).

### 1.2 Controlled light stress application to enhance morphological and nutritional plant quality

Morphological and nutritional enhancements in plants can result from defensive mechanisms effected in response to stress conditions caused by high light energy. For instance, compaction of lettuce rosette, which could be considered as a desirable morphological trait for consumers, occurs in response to increased radiative energy allowing for the regulation of light capture and prevention of photo-damage (Cammarisano et al., 2021). Likewise, leaf thickness, which increases in response to light intensity and spectrum (Kang et al., 2014; Cioć and Pawłowska, 2020), can represent a demanded trait for marketability and for improved shelf life of lettuce (Clarkson et al., 2003). And, similarly, antioxidants, e.g., β-carotene and phenolics, represent effective protective compounds against oxidative stress both in plants (Dumanovic et al., 2020) and humans (Sharifi-Rad et al., 2020). Another plant bioactive compound, which rapidly fluctuates in response to light, with stimulation by excess light energy (Havaux et al., 2004), and which has been proved to have beneficial effect on human eye and brain health, is zeaxanthin (Demmig-Adams and Adams, 1992). Increased radiative energy effects can be triggered by light intensity and/or by high energy photons, i.e. short wavebands photons (Cammarisano et al., 2020). Light intensity has stimulating effect on antioxidant compounds (Sutuliene et al., 2022). In fact, light treatment with increased intensity applied at the end-of-production is proposed as a strategies to enhance nutritional quality of basil (Larsen et al., 2022) and lettuce (Gómez and Jiménez, 2020) indoor farming. The use of higher energy wavebands such as blue triggers plant photomorphogenic responses and allows the stimulation of secondary metabolites synthesis (Jung et al., 2021); thence, increasing plant morphological and nutritional quality. Blue radiation has been reported as highly effective in promoting accumulation of chlorophylls, carotenoids and anthocyanins in several plant species (Johkan et al., 2010; Xu et al., 2014; Taulavuori et al., 2016; Frede and Baldermann, 2022; Liu et al., 2022). Stress conditions can be beneficial for increasing plant defensive strategies, which in some cases are considered quality traits by consumers, i.e. antioxidants, though when the stress exceeds certain tolerance thresholds plant biomass could be negatively affected (Zhang et al., 2020).

### 1.3 Detection of changes in leaf biochemistry to monitor plant performance and quality

Detectable changes of compounds synthesized by the plant in response to stress could be used to monitor stress extent and hence, the balance between plant performance and nutritional quality in order to obtain predictable quality yield. Compounds such as phenolics, e.g. anthocyanins, which are considered to be beneficial to human health (Sen and Chakraborty, 2011) and which increase in presence of excessive radiative energy (Demmig-Adams and Adams, 1992), could be used as markers to monitor nutritional quality in terms of antioxidant content. Besides antioxidants, photosynthetic pigments, i.e., chlorophylls (chl) and carotenoids (car), can be indicative of plant performance (Ling et al., 2011; Croft et al., 2017). Total chlorophyll content (chl a+b) and the ratio between chlorophylls a and b (chl a/b) positively correlate with photosynthetic capacity of plants (Croft et al., 2017) and are adjusted to the surrounding environment to enhance plant growth. For instance, chl a/b increases with higher light intensity as strategy to intensify light absorption of photosystem I in order to achieve a more balanced excitation state (Hogewoning et al., 2012). Content of photosynthetic and photoprotective pigments is rapidly adjusted by the plant in response to the environment and could be used as markers in noninvasive procedures, which would allow instant monitoring of plant nutritional quality adjustments and early warning for stress through spectroscopy and radiation transfer models (Blackburn, 2007).

Most non-destructive techniques to measure leaf biochemical composition found in literature are based on optical spectroscopy. The latter allows to measure the interaction of matter with electromagnetic radiation and has been used for studying properties of all kinds of materials for centuries (Baudelet, 2014). In plant research, such technique is widely proposed for remote sensing of physiological traits of forests and agricultural fields. Several studies propose leaf spectroscopy to detect, with different degree of spatial resolution, e.g. leaf to canopy, based on the specific technology, content of water (Ceccato et al., 2001), nitrogen (Wang et al., 2016), pigments (Blackburn, 2007), secondary compounds (Jayapal et al., 2022) and signs of abiotic (Grzesiak et al., 2009) and biotic (Mahlein et al., 2012) stresses (Jacquemoud and Ustin, 2001).

Leaf spectroscopy combined with physical based leaf radiation transfer models, with given technical adaptations, could be used to monitor leaf pigments in real-time and within the growth environment, giving the possibility to adjust the micro-climate in case of stress detection or harvest the crop at the exact quality status. Ideally, dynamic biochemical changes influenced by various abiotic parameters, e.g. spectral light intensity, temperature, humidity, and their combined effects, should be characterised in order to define pigment dynamics for each stress type and their combination to be used as target values for controlling plant growth and nutritional quality in indoor farming.

In this study, we aimed at 1) inducing leaf pigment changes by using light intensity and spectral composition, 2) characterising these changes during light treatment duration, and 3) developing a non-destructive procedure to detect the dynamic changes. For that, we applied a blue wavelength enhanced white light spectrum at two photon flux densities (PFDs) for 14 days on a typical green lettuce cultivar and a lettuce cultivar with strong red coloration from anthocyanins and determined leaf pigments including chlorophylls, carotenoids and anthocyanins in response to light in time.

## 2 Materials and methods

### 2.1 Experimental design

The experiment was executed at the Leibniz-Institute of Vegetable and Ornamental Crops IGZ (Grossbeeren, Germany) in a growth chamber with controlled atmosphere regarding temperature and relative humidity. The experimental system consisted of two double-layer shelving units providing four growing areas of 0.27 m^2^ each (separated by white reflective plastic sheets). Each layer was equipped with two dimmable LEDs arrays (LightDNA8, Valoya, Finland) and hosted twelve plants, six per cultivar (plant density of 44.4 plants m^-2^). Two light spectral treatments (white light spectrum with 15% blue (WB15) and with 40% blue (WB40)) were tested at two photon flux densities, PFDs, (160 and 240 µmol m^-2^ s^-1^). The resulting four light treatments (160WB15, 160WB40, 240WB15, 240WB40; cf. **Table 1**) were applied on two lettuce cultivars (green leaf lettuce ‘Aquino’ cv. (CV_g_), red leaf lettuce ‘Barlach’ cv. (CV_r_), Rijk Zwaan, The Netherlands). The two light spectra were simultaneously tested in two distinct growing compartments of the chamber each (i.e. four compartments were used), and the experiment was repeated twice (sowing on 26 January and 7 March 2022) at 160 µmol m^-2^ s^-1^ and twice (sowing on 27 December 2021 and 7 April 2022) at 240 µmol m^-2^ s^-1^.

**Table 1:** Treatment codes and their spectral composition (in percentage) for the four light treatments: White light control and white-blue light at 160 (160WB15, 160WB240) and at 240 (240WB15, 240WB40) µmol m^-2^ s^-1^.

Treatment code	Waveband percentage		PFD, µmol m^-2^ s^-1^
	White [Green:Red:FR]	Blue	
160WB15	75 [40:29:16]	15	160
160WB40	60 [35:16:9]	40	160
240WB15	75 [40:29:16]	15	240
240WB40	60 [35:16:9]	40	240

Blue (400-480 nm, with peaks at 420 & 450 nm), Green (481-599 nm), Red (600-669 nm), FR (670-780 nm).

### 2.2 Plant growth conditions and light treatments

Seeds of each of the two lettuce cultivars ‘Barlach’ (CV_r_) and ‘Aquino’ (CV_g_) were sown in stone-wool cubes (4 cm, Rockwool^®^, Grodan, Roermond, The Netherlands) and kept at 4°C in the dark for 24 hours. Afterwards, the cubes were moved to the experimental unit and kept under white light spectrum (PFD of 160 µmol m^-2^ s^-1^ and photoperiod of 18 h) for 18 days. Temperature and relative humidity were controlled to 20°C (day and night) and 60% (day and night), respectively, for the whole duration of the experiments. Controlled environmental parameters were monitored (logging every 5 minutes) within each growing area (Tinytag Ultra 2, Gemini Data Loggers, Chichester, UK). Growing areas were automatically irrigated for 1 minute five times a day, using lettuce nutrient solution (Sonneveld and Straver, 1994) (EC: 1.9 dS m^−1^, pH: 5.5–6). On day 18, 48 homogeneously germinated plants (≥ 6 expanded leaves) were selected from each of the two cultivars, including the substrate, were embedded into larger stone-wool cubes (10 cm, Rockwool^®^, Grodan, The Netherlands) and positioned onto the four growing areas (12 in each). From day 19, light treatments (160WB15, 160WB40, 240WB15, 240WB40) were applied for the following 14 days. The two spectral light treatments were composed of four main channels: blue (400-480 nm), green (481-599 nm), red (600-669 nm) and far-red (670-800 nm). WB15 resulted in 15% blue, 40% green, 29% red, 16% far red. WB40 resulted in 40% blue, 35% green, 16% red, 9% far-red, (**Table 1**). Spectral light intensity (PFD) was measured at canopy level (distance from lamps: 40 cm) using a spectroradiometer (UPRtek PG200N, 350–800 nm; UPRtek Corp., Taiwan).

### 2.3 Plant measurements

**Figure 1:**
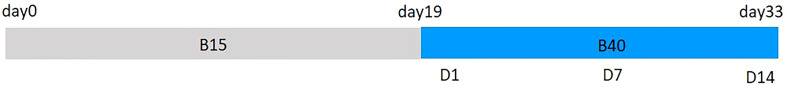
Scheduled growth periods of lettuce plants from sowing date (day 0) to harvest day (day 33). Plants were grown under white light spectrum (B15) for 18 days and treated with blue-enriched spectrum (B40) from day 19 for the following 14 days. Plant measurements were performed at 1 day, 7 days and 14 days after treatment start.

Time course measurements of the same plant samples were performed at three points during treatment application, one day (D1), seven days (D7) and fourteen days (14D) after treatments begun (**Figure 1**). Chlorophyll *a* fluorescence measurements and assessment of leaf pigments, through destructive (*in vitro*) and non-destructive (*in vivo*) spectrophotometry, were performed. Same leaf was used both for estimating pigment content through optical measurements followed by mathematical modeling and pigment quantification through laboratory procedure. Pigment extraction and quantification was done for total chlorophylls and carotenoids, while pigment estimation was feasible for anthocyanins, chlorophylls, carotenoids and their zeaxanthin fraction. Measurements started with excising one leaf for optical readings, followed by sampling of the same leaf in liquid nitrogen for subsequent pigments extraction and quantification. Assessed leaf area for optical properties was approx. 1 cm^2^, while the whole leaf was sampled for pigment extraction. Leaf numbers 6, 9 and 12 were chosen for leaf measurements on D1, D7 and D14, respectively, being the youngest and most expanded ones. At the same time, the rest of the plant was measured for chlorophyll *a* fluorescence. Same plant was measured on the three sampling dates. On each sampling date, twenty-four plants were measured from 9:00 a.m. to 1:00 p.m. Consecutive measurements were alternated between cultivars and light treatments accordingly.

#### 2.3.1 Measurements of leaf optical properties

*In-vivo* leaf spectroscopy was used to measure leaf optical properties. Immediately after excision, each leaf was assessed on the two sides of the midrib (adaxial side) for reflectance and transmittance using a double-beam spectrophotometer (V-670, Jasco, Japan) equipped with an internal integration sphere (ILN-925, inner diameter: 150 mm). Measuring range was set between 340 and 900 nm. Each instrument scan over this range required less than 2 minutes. A certified white reflective standard was used to calibrate reflectance readings.

#### 2.3.2 Estimation of leaf pigments from leaf reflectance and transmittance

Using the leaf spectral models PROSPECT-D and Fluspect-CX, we estimated several leaf properties using least squares minimization of the difference between PROSPECT-D predicted and measured reflectances and transmittances (400 nm - 900 nm). Among the estimated leaf properties were the leaf content of chlorophyll, carotenoid and anthocyanin as well as the zeaxanthin+antheraxanthin to total xanthophyll ratio. Least square minimization was done using a trust region algorithm (fmincon matlab function, 1990-2019, The MathWorks, Inc.) with box-constraints on parameters (i.e. leaf properties) and an additional constraint on the chlorophyll to carotenoid ratio (4.4 < chl/car < 6.4), which corresponds to the observed range from lab analysis (see next section).

#### 2.3.3 Extraction and quantification of leaf pigments

Chlorophyll and carotenoid content of leaves was determined through the wet chemistry procedure reported in (Cammarisano et al., 2021). Prior extraction, leaf samples were freeze-dried for 5 days and ground to fine powder. For the extraction, 1.6 – 2.8 mg of leaf tissue powder was weighed in triplicates in 2 ml green centrifuge tubes. 0.6 ml of 95% ethanol were added to each tube. After being vortexed and left in the fridge for the consecutive 24 hours, tubes were centrifuged and extracts were collected. The same extraction procedure was repeated 3 times, with three washes (0.6, 0.6 and 0.5 ml, respectively) in three following days. The combined extract for each sample was then read (at 470, 649, and 664 nm) in triplicates against the same amount (170 µl) of blank solution using a UV/VIS spectrophotometer (Infinite M200PRO, Tecan, Switzerland). Samples were measured in a 96-well half area microplate (UV-STAR, flat bottom, Greiner bio-one Gmbh, Austria), which was used to ensure a 1 cm pathlength. Chlorophylls a and b and total carotenoids concentration was determined using equations reported in Lichtenthaler and Buschmann (2001).

#### 2.3.4 Measurements of chlorophyll *a* fluorescence

Light adapted plants were measured for chlorophyll a fluorescence using the modulated fluorescence imaging FluorCam system (Photon Systems Instruments, Brno, Czech Republic). Shutter time and sensitivity of the charge-coupled device (CCD) camera were chosen based on a spare sample and kept the same for all measurements. The distance between the camera lens and the upper leaves was maintained at 24 cm. A horizontal spot of 5380 pixels was used on the youngest and most expanded leaf to ensure comparable assessed area between measured plants. Calculated parameters are given in the **Table 2**.

**Table 2:** Chlorophyll *a* fluorescence parameters (steady state quantum yield, QY, φPSII; Lichtenberger vitality index, Rfd; energy-dependent quenching, qE) assessed 1, 7 and 14 days after high blue light treatment start on two lettuce cultivars, cv. ‘Aquino’ and cv. ‘Barlach’.

Parameter	Formula	Description
QY, φPSII	(F_M__Lss - F_t__Lss)/F_M__Lss*	Steady state Quantum Yield
Rfd	(F_P_ - F_t__Lss)/F_t__Lss*	Lichtenberger vitality index
qE	(F_M__D3 - F_M__Lss)/F_M__D3*	Energy-dependent quenching

*where F_M__Lss is the steady-state maximum fluorescence in light, F_t__Lss is the steady-state fluorescence in light, F_p_ is the peak fluorescence during the initial phase of the Kautsky effect, F_M__D3 is the instantaneous maximum fluorescence during dark relaxation.

### 2.4 Plant growth

Fresh and dry weight of the lettuce rosettes were determined at 14 days after treatment start, which coincided with the end of the experiment, following procedure described by (Cammarisano et al., 2021). A total of six intact plants per spectral light intensity treatment were harvested.

### 2.5 Data processing and statistics

Data were processed and statistically analyzed using Microsoft Excel Standard 2013 and R studio (R version 2022.02.2, “Prairie Trillium”) with package “agricolae” (Mendiburu, 2010). Repeated measures ANOVA could not be used as sample size between different measuring dates was uneven. Three-way ANOVA was used to determine whether duration of treatment, light spectrum, PFD and their interactions significantly (p ≤ 0.05) affected the measured response variables. Each cultivar was separately analysed. All determined variables (estimated chlorophylls, carotenoids and anthocyanins, lab quantified chlorophylls and carotenoids, chlorophyll a fluorescence parameters) were individually analysed. Two-way ANOVA was used for fresh and dry weights of lettuce heads to test for the effect of light spectrum and PFD. Least Significance Difference (LSD) was used as *post hoc* test to localize the differences between means at 5% significance level. Student’s t-test was then performed at individual measuring days to locate specific means where the light spectrum had a significant effect. Correlation between modelled and quantified chlorophylls and carotenoids was performed after converting the area based estimated pigment content to dry mass based content.

## 3 Results

### 3.1 Leaf pigments

#### 3.1.1 Leaf pigment content

**Figure 2:**
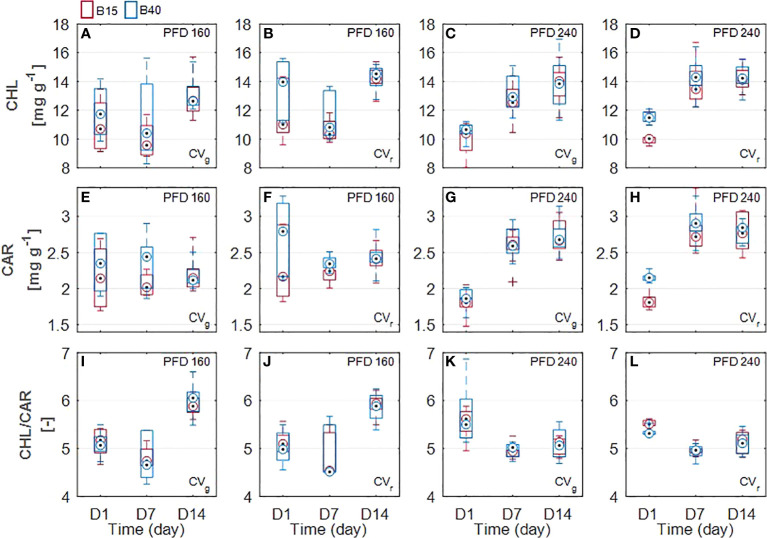
Measured leaf pigments of two lettuce cultivars (Aquino cv., CV_g_ and Barlach cv., CV_r_) grown under two photon flux densities (PFDs; 160 and 240 µmol m^-2^ s^-1^) and treated with two light spectra (white light spectrum with 15% blue, B15, in red; white light spectrum with 40% blue, B40, in blue) for 14 days. Pigment content was determined over three time points (day 1, day 7, and day 14 after treatment start) for total chlorophylls **(A-D)**, total carotenoids **(E-H)**, and chlorophyll/carotenoid ratio **(I-L)**. Boxplot whisker (W) was set to 2.5, and points were drawn as outliers if they were larger than Q25+W(Q75-Q25) or smaller than Q25-W(Q75-Q25).

The increased percentage of blue radiation (B40) used in the study significantly affected the leaf pigments quantified through wet chemistry procedure in the red-leaf lettuce cultivar samples. Both chlorophyll and carotenoid content was found to be significantly greater in leaves of CV_r_ treated with B40 compared to B15 samples. In particular, statistical increase in chlorophylls was detected at D1 both in 160WB40 (13%) and 240WB40 (12%) red-leaf samples (p = 0.03 and p = 7x10^-6^, respectively). A general increasing trend in chlorophyll content over the treatment duration was observed in both cultivars at higher PFD. Total carotenoid content resulted significantly greater in CV_r_ leaves at D1 (15%) and D7 (7%) in 160WB40 samples and only at D1 (15%) in 240WB40 samples (**Figures 2E–H**). At higher PFD (240), carotenoids tended to generally increase over time (from D1 to D14) in both lettuce cultivars. For both cultivars, the chl/car ratio was significantly different under the two tested PFD levels, 160 and 240 µmol m^-2^ s^-1^, showing opposite behavior. If under lower PFD the greatest chl:car ratio was detected at D14, the greatest ratio under the highest PFD was detected at D1 (**Figures 2I–L**).

#### 3.1.2 Leaf pigment estimation

**Figure 3:**
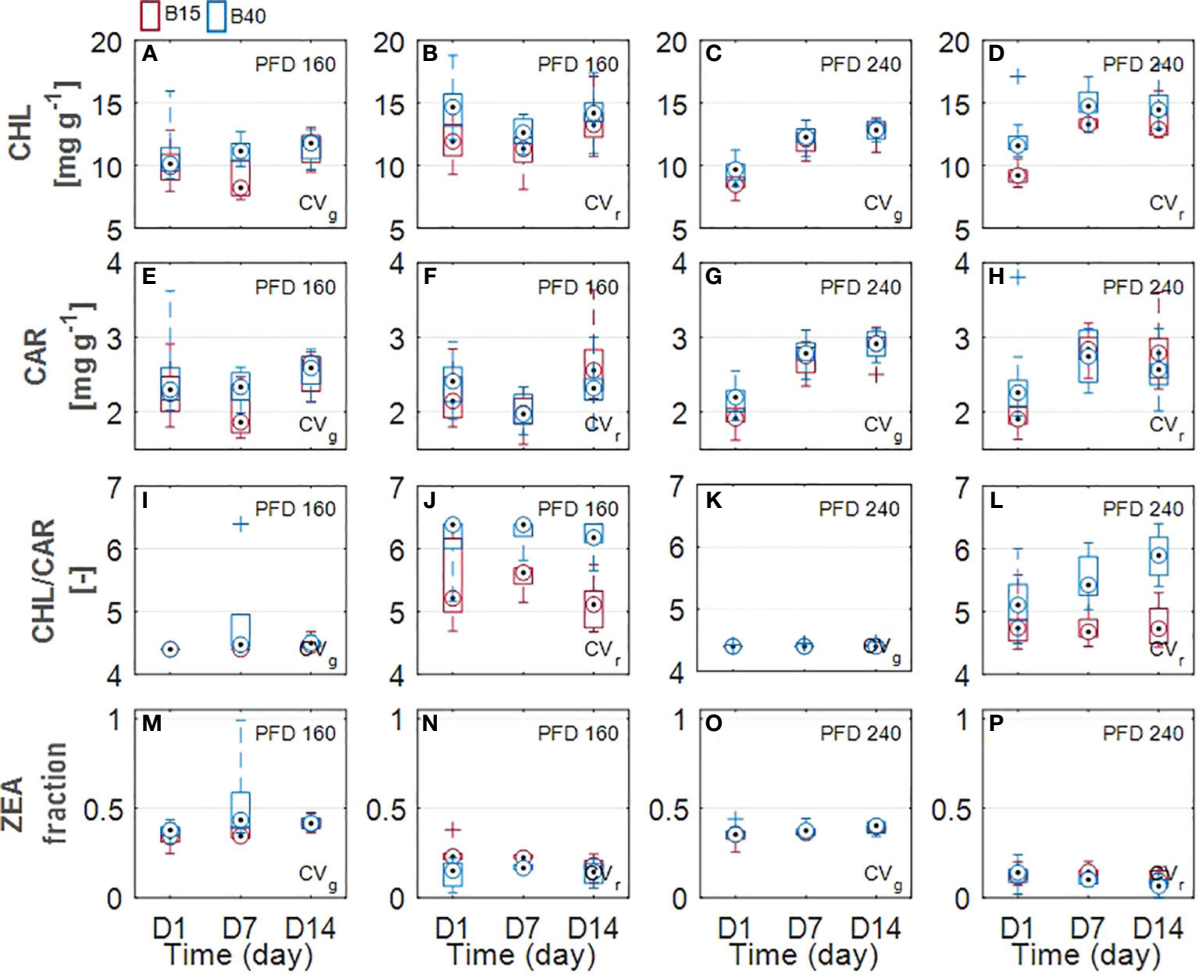
Estimated leaf pigments of two lettuce cultivars (Aquino cv., CV_g_ and Barlach cv., CV_r_) grown under two photon flux densities (PFDs; 160 and 240 µmol m^-2^ s^-1^) and treated with two light spectra (white light spectrum with 15% blue, B15, in red; white light spectrum with 40% blue, B40, in blue) for 14 days. Pigment content was determined at three time points (day 1, day 7, and day 14 after treatment start) for total chlorophylls **(A–D)**, total carotenoids **(E–H)**, chlorophyll-carotenoid ratio **(I–L)**, and fraction zeaxanthin **(M–P)**. Boxplot whisker (W) was set to 2.5, and points were drawn as outliers if they were larger than Q25+W(Q75-Q25) or smaller than Q25-W(Q75-Q25).

**Figure 4:**
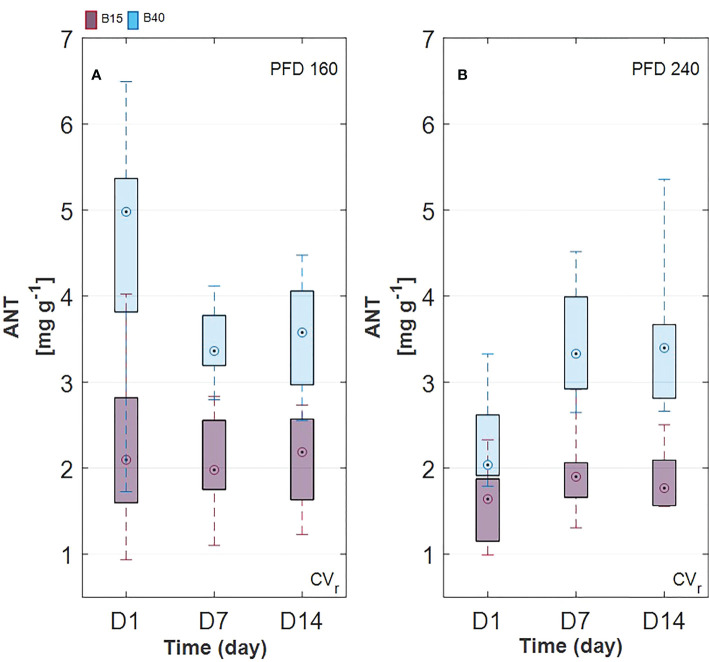
Estimated anthocyanin content in Barlach cv. (CV_r_) grown under two photon flux densities (PFDs; 160 **(A)** and 240 **(B)** µmol m^-2^ s^-1^) and treated with two light spectra (white light spectrum with 15% blue, B15, in red; white light spectrum with 40% blue, B40, in blue) for 14 days. Pigment content was determined over three time points (day 1, day 7, and day 14 after treatment start). Boxplot whisker (W) was set to 2.5, and points were drawn as outliers if they were larger than Q25+W(Q75-Q25) or smaller than Q25-W(Q75-Q25).

Estimated leaf pigment was significantly affected by the increased percentage of blue radiation (B40) in the two investigated lettuce cultivars (**Figures 2**, **3**). Estimated leaf pigments showed similar behavior to that of measured ones. In addition to chlorophylls and carotenoids, zeaxanthin fraction, defined as (Z+A)/(Z+A+V), and anthocyanins were estimated. In CV_r_, the estimated zeaxanthin fraction was significantly decreased by blue enriched light spectrum (B40, **Figures 3M–P**) at lower PFD, while it was increased by B40 at 240 µmol m^-2^ s^-1^ (27 to 47% increase from D1 to D14 in 160B40 compared to 240B15). Estimated anthocyanins were only detectable in CV_r_, in which, they were increased by light spectrum (B40) with stronger effect at lower PFD (maximum of 50% increase at 160 µmol m^-2^ s^-1^ and 19% at 240 µmol m^-2^ s^-1^) (**Figure 4**).

#### 3.1.3 Correlation between estimated and quantified leaf pigments

**Figure 5:**
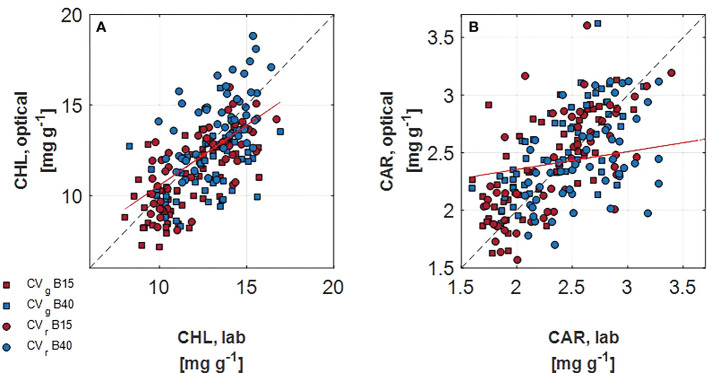
Correlation of lab measured and optically assessed total chlorophylls **(A)** and total carotenoids **(B)** for two lettuce cultivars (Aquino cv., CV_g_, squares, and Barlach cv., CV_r_, circles) treated with two light spectra (white light spectrum with 15% blue, in red, B15; white light spectrum with 40% blue, in blue, B40) after 14 days of treatment. The red line indicated the regression line over all data points. The 1:1 response is indicated as dashed line.

In spite of the similar ranges of chlorophylls and carotenoids observed in the measured and the estimated leaf pigments, the correlation analysis determined low association between the two procedures. Overall, greater correlation was detected for chlorophylls than carotenoids (**Figure 5**). In addition, correlation coefficient was very variable when grouping the different levels of each factor. For instance, when considering chlorophylls, greatest correlation coefficients were found for B15 (r = 0.72) and for CV_r_ (r = 0.70). A lowering of correlation coefficients was observed with the progress of the treatment duration, with r decreasing from 0.68 for day 1 and 7 to 0.34 for day 14.

### 3.2 Chlorophyll *a* fluorescence

**Figure 6:**
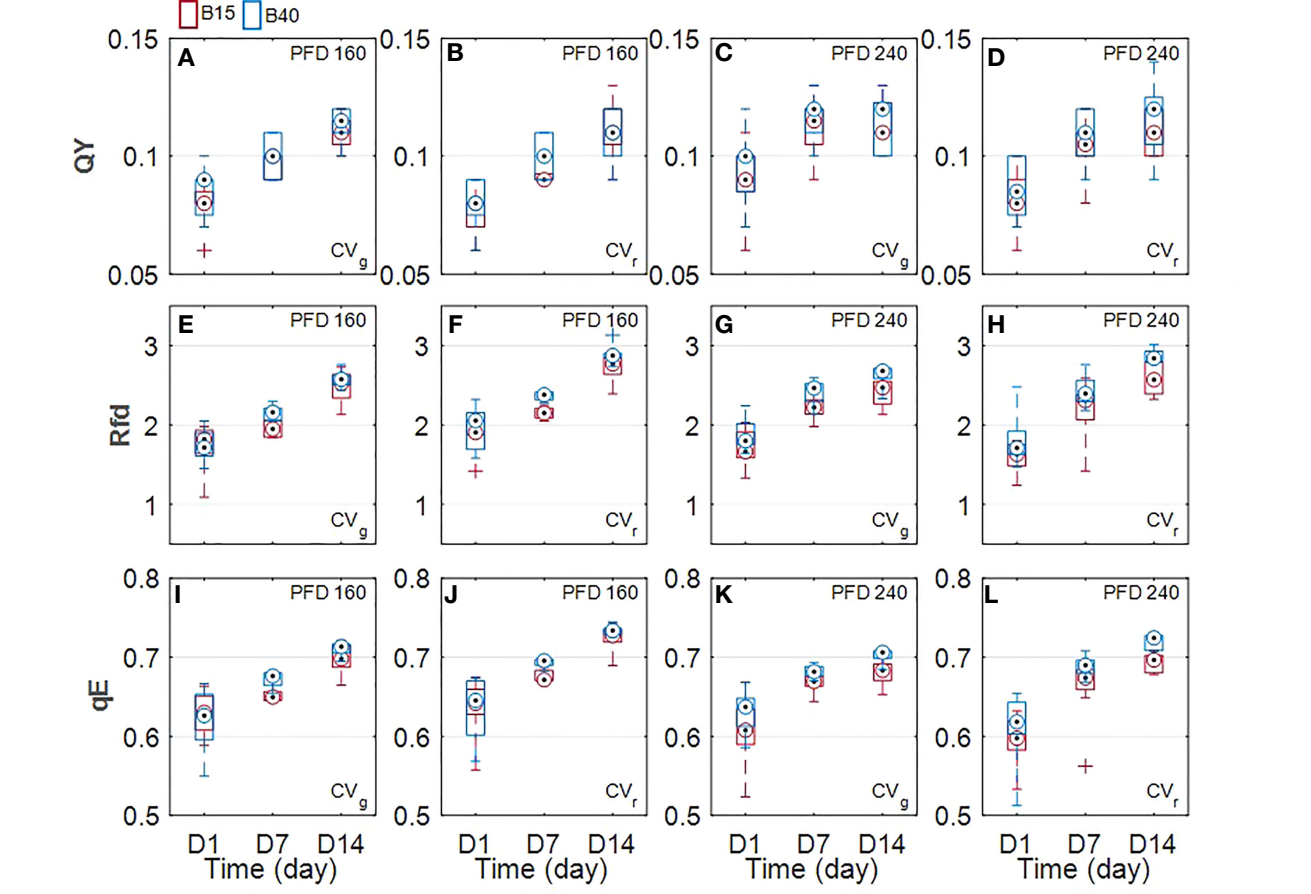
Measured light-adapted chlorophyll *a* fluorescence parameters 1. steady state quantum yield (QY; φPSII; **A-D**), 2. Lichtenberger vitality index (Rfd; **E-H**), and 3. energy-dependent quenching (qE, **I-L**) of two lettuce cultivars (Aquino cv., CV_g_ and Barlach cv., CV_r_) grown under two photon flux densities (PFDs; 160 and 240 µmol m^-2^ s^-1^) and treated with two light spectra (white light spectrum with 15% blue, B15, in red; white light spectrum with 40% blue, B40, in blue) for 14 days. Chlorophyll *a* fluorescence was determined over three time points (day 1, day 7, and day 14 after treatment start). Boxplot whisker (W) was set to 2.5, and points were drawn as outliers if they were larger than Q25+W(Q75-Q25) or smaller than Q25-W(Q75-Q25).

All measured chlorophyll *a* fluorescence parameters tended to increase with time in all analysed samples (**Figure 6**). QY was not significantly affected by the light treatments. Statistically greater Rfd and qE values were identified between samples treated with differing percentage of blue radiation. Greatest Rfd and qE occurred in B40 samples at D7 and D14, with same behavior observed for both used lettuce cultivars and PFDs. Only exception was the CV_g_ under the lower PFD which did not show any significant effect of light spectrum (**Figure 6**).

### 3.3 Plant growth

Fresh and dry weights of the lettuce rosette were significantly affected by PFD in both cultivars (**Table 3**). Fresh weight was increased of 56% for CV_g_ and of 42% for CV_r_ under the higher PFD, compared to the lower one. Significantly lower dry weight (-23%) was observed for lettuce heads of CV_g_ treated with increased percentage of blue radiation (B40) compared to control rosettes.

**Table 3:** Growth response (fresh and dry weights determined 33 days after sowing) of two lettuce cultivars (cv. Aquino, CV_g_ and cv. Barlach, CV_r_) treated for the last 14 days with blue-enriched white light spectrum (B40) compared to plants grown under white light spectrum (B15).

Treatments	Fresh weight (g) (g/head)	Dry weight (g) (g/head)
*Cultivar Aquino*, CV_g_		
160B15	11.14 ± 0.64^b^	0.63 ± 0.03^b^
160B40	10.18 ± 0.28^b^	0.49 ± 0.05^c^
240B15	24.89 ± 2.24^a^	1.16 ± 0.09^a^
240B40	23.08 ± 1.85^a^	1.07 ± 0.07^a^
*Cultivar Barlach*, CV_r_		
160B15	14.72 ± 1.14^b^	0.83 ± 0.06^b^
160B40	13.53 ± 0.51^b^	0.77 ± 0.02^b^
240B15	25.83 ± 1.98^a^	1.17 ± 0.07^a^
240B40	22.95 ± 1.98^a^	1.07 ± 0.08^a^

Values are reported as mean ± standard error. Different letters within columns indicate significant treatment differences at P < 0.05, as determined by Fisher’s least significant difference (LSD) test, with a > b > c. Light spectral treatments were applied at two PFDs of 160 and 240 µmol m^-2^ s^-1^. Tested factors were PFD, blue percentage and their interactions. Only PFD was significant at p < 0.001.

## 4 Discussion

The estimation of leaf pigments through non-destructive techniques such as the combination of leaf optical measurements and PROSPECT-D model inversion used in this study could represent a potential method for the *in vivo* monitoring of leaf biochemical changes indicative of plant abiotic stress progress and relative nutritional quality improvements. Parameters indicative of a rise in the plant stress level in response to light, as for instance the increasing ratio of zeaxanthin over carotenoids (Xie et al., 2020) and anthocyanins over chlorophylls (Kim et al., 2012; Zheng et al., 2021), could be used both for detecting exacerbation of stress response and increase of bioactive compounds. In contrast to strategies using portable leaf reflectance meter (Jiménez-Lao et al., 2021), this technique offers potentials for continuous monitoring of plant pigment content within the growth environment for instance by using hyperspectral cameras (Jayapal et al., 2022). The advantage of *in vivo* monitoring of leaf pigments is essential for detection of the dynamic plant response in adjusting the content of these compounds in response to the surrounding environment. Indeed, content of pigments, including chlorophylls, carotenoids and flavonoids, has been found to vary according to the radiation dose and duration. For instance, applying low dose of UV-B radiation for 30 minutes to basil caused a fast decrease in zeaxanthin content followed by a strong increase in the next 48 hours during recovery time. When applying high dose of UV-B, zeaxanthin content decreased continuously from the first minutes of application to reach values close to zero after 24 hours (Mosadegh et al., 2019).

**Figure 7:**
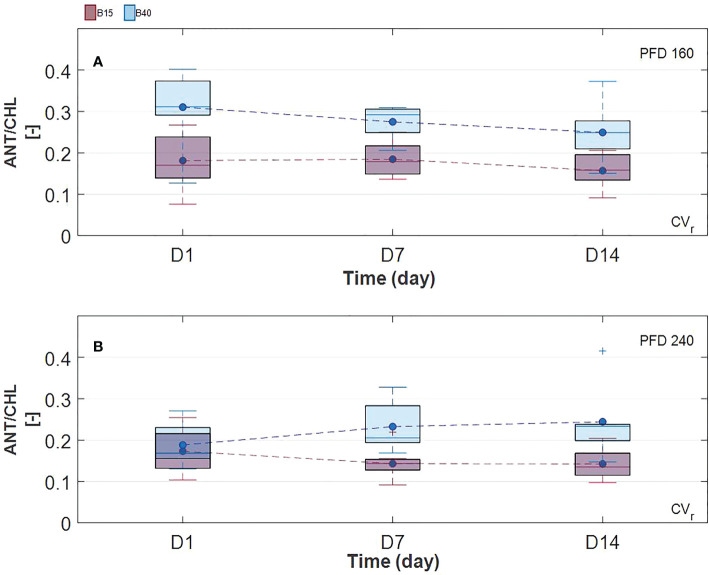
Ratio of estimated anthocyanins over chlorophylls, ANT/CHL, in time in Barlach cv. (CV_r_). Plants were grown under two photon flux densities (PFDs; 160, **A**, and 240, **B**, µmol m^-2^ s^-1^) and treated with two light spectra (white light spectrum with 15% blue, B15, in red; white light spectrum with 40% blue, B40, in blue) for the last 14 days of growth. Pigment content was determined over three time points (day 1, day 7, and day 14 after treatment start).

Chlorophylls have a key role in photosynthesis and their content has been found to change in response to various stresses (Muhammad et al., 2020), with rising behaviour under low-level stress and decreasing behaviour under more severe stress (Agathokleous and Penuelas, 2020). Otherwise, carotenoids and anthocyanins, which can be considered as protective pigments for their photoprotective and antioxidant properties, show increasing response to several stresses (Chalker-Scott, 1999; Havaux, 2014). From the results obtained in the current study, we could observe a pigment response to light spectrum with increased blue radiation (B40) which was similar between measured and estimated pigments (**Figures 2**, **3**). Total chlorophylls and carotenoids were significantly greater in B40 but only during the first days of treatment application. When considering the ratio of chl/car though, a dual behaviour could be observed. While at lower PFD (160 µmol m^-2^ s^-1^) the greatest chl/car ratio was detected in B40 samples at D14, at higher PFD (240 µmol m^-2^ s^-1^) the greatest chl/car ratio was at D1 followed by constant decline with treatment duration. Such opposite trend could confirm the response of chlorophylls to mild versus more severe stress reported in literature (Agathokleous and Penuelas, 2020). At low PFD, the plant stress level might have been low enough to allow for mitigation through increased potential for light use (> chlorophylls). The higher PFD was probably close to the threshold tolerance range for the plant, which invested in increasing protective pigments (> carotenoids) to acclimate to the extended exposure to B40 light treatment. Likewise, CV_r_ invested in anthocyanins over the duration of 240B40, with the ratio ANT/CHL rising over time meaning that the net rate of anthocyanins biosynthesis was increasing over that of chlorophylls (**Figure 7**). However, PFD of 240 µmol m^-2^ s^-1^ was far from the maximum saturating light level (400-700 µmol m^-2^ s^-1^) reported for lettuce cultivated at temperatures of 20°C (Weigou et al., 2012; Cammarisano et al., 2020; Zhou et al., 2022). Indeed, the optimal vitality index (Rfd, **Figures 6E-H**), which expresses the potential photosynthetic activity of PSII, was never below the critical value of 1.0 (Haitz and Lichtenthaler, 1988). Rather it constantly raised reaching values indicative of high photosynthetic efficiency (< 3.0) at D14 in all treatments, with relative greater increase in response to higher PFD and to greater percentage of blue light. The observed pigment dual behaviour under the two tested PFDs and their dynamics highlight the significant need of characterising pigment changes to 1) varying climate and 2) over time.

Though, the trend in the pigment dynamics was similar between the two monitoring procedures, the absolute values did not match precisely (**Figure 5**). The r coefficient tended to lower with time (D1+D7>D14).This could be due to the fact that later measuring time corresponds to much larger portions of leaf sampled for lab analysis (the whole leaf was sampled, including veins and leaf area not uniformly exposed to the light) compared to portions used for optical measurements (a standard area of 1 cm^2^ of leaf tips was used for optical readings). In addition, it needs to be considered that varied correlation between differently aged leaves may be expected as leaf structure is modified with leaf growth and consequently leaf spectral properties change (Gara et al., 2019). PROSPECT versions for estimating pigments have been optimised during the years, going from exclusively considering chlorophylls, to inclusion of carotenoids with PROSPECT-5 (Feret et al., 2008) and addition of anthocyanins with PROSPECT-D (Féret et al., 2017). Further improvements of PROSPECT model inversion allow to estimate leaf biochemical composition with sole use of reflectance or transmittance data (Sun et al., 2018). Future attempts to improve correlation could regard the sampling procedure by exactly matching the leaf portion used for optical measurement to that used for quantifying pigments. In this study, in order to match units, area based pigment estimates obtained by PROSPECT-D where converted to mass based estimates using independent estimates of leaf mass per area, which are usually known at best at the treatment level and not for each leaf spot. Unexpectedly, correlation for CV_r_ resulted to be greater compared to CV_g_ (**Figure 5**). Such result endorses the use of physical based models like PROSPECT-D under more general conditions, i.e. having low and high anthocyanin containing cultivars without prior calibration (Féret et al., 2017) which would be required with empirical regression models.

Additionally, species and cultivars differences should be taken into account. In fact, variability in pigments and their ratios can be very high between plant species and also between cultivars of the same plant species as reported in (Kowalczyk et al., 2016; Mastilović et al., 2020), where different cultivars of lettuce grown under the same conditions showed significant variability in chlorophyll and carotenoid content. In our study, distinct response of the two lettuce cultivars to light spectral intensity was observed. Most of the significant pigment changes were spotted in CV_r_, confirming its greater adaptability to light compared to green leaf cultivars. In fact, no negative effect of the B40 treatment was observed in fresh and dry weight of CV_r_, instead dry weight of CV_g_ was statistically diminished under B40 suggesting a less tolerating nature toward this light treatment. These results agree with the study of (Frąszczak and Kula-Maximenko, 2021), where red leaf lettuce cultivars accumulated more biomass and performed better compared to green leaf cultivars under blue-rich light spectrum. Due to the observed divergence in pigment changes between CV_g_ and CV_r_, future works should address solutions to the issue of cultivar- and species- specific responses when defining stress indicators. For instance, the increase in the ANT/CHL ratio could be suggested as a stress versus quality indicator specific for red leaf cultivars (**Figure 7**).

Another important discussion point regards how to adapt spectra acquisition hardware and software for such a monitoring system for practical conditions, e.g. using PROSPECT-D with imaging data (Procosine), (Jay et al., 2016) and optimized settings for retrieval if only reflectance data are available (Spafford et al., 2021).

## 5 Conclusion

The current study proposes the combination of leaf optical measurements and PROSPECT-D model inversion as a potential approach for the *in vivo* monitoring of dynamic leaf biochemical changes indicative of plant abiotic stress and relative nutritional quality improvements in indoor farming. Still, there is some necessary progress to consider in future works for the development of such a system, including 1) improvement of the correlation between measured and estimated pigments to exactly estimate leaf pigment content, 2) study of the pigment responses in various plant species and cultivars to help characterising the range of responses, and 3) investigation of the responses to varying climate and nutrient solutions in order to determine stress type effects. A potential solution to develop an effective monitoring system would be to combine different detection methods, e.g. spectroscopy, imaging, thermography, fluorescence, to facilitate the distinction of species- and stress type- influence. And, connecting such sensing systems to a decision support system would allow the automated adjustment of the environment according to what is needed to reach the predicted target produce yield and nutritional content, additionally contributing to increase of resource use efficiency by more accurate match between plant needs and resource application.

## Data availability statement

The original contributions presented in the study are included in the article/supplementary material. Further inquiries can be directed to the corresponding author.

## Author contributions

LC, JG, and OK contributed to conception and design of the study. LC performed the experimental investigation, LC, JG cured data, OK cured data visualization, LC performed the statistical analysis and wrote the first draft of the manuscript, LC, OK and JG wrote sections of the manuscript. OK acquired funding. All authors contributed to the article and approved the submitted version.

## Conflict of interest

The authors declare that the research was conducted in the absence of any commercial or financial relationships that could be construed as a potential conflict of interest.

## Publisher’s note

All claims expressed in this article are solely those of the authors and do not necessarily represent those of their affiliated organizations, or those of the publisher, the editors and the reviewers. Any product that may be evaluated in this article, or claim that may be made by its manufacturer, is not guaranteed or endorsed by the publisher.
